# More than half of systematic reviews have relevant core outcome sets

**DOI:** 10.1016/j.jclinepi.2021.04.019

**Published:** 2021-08

**Authors:** Ian J. Saldanha, Susanna Dodd, Sarah L. Gorst, Paula R. Williamson

**Affiliations:** a Center for Evidence Synthesis in Health, Department of Health Services, Policy, and Practice (Primary), Department of Epidemiology (Secondary), Brown University School of Public Health, Providence, Rhode Island, USA; b MRC/NIHR Trials Methodology Research Partnership, Department of Health Data Science, University of Liverpool (a member of Liverpool Health Partners), Liverpool, UK

**Keywords:** Core outcome sets, Outcomes, Systematic reviews, Matching, Scope, Relevance

## Abstract

**Objectives:**

Using recent systematic reviews (SRs), our objectives were to: (1) develop a framework to assess whether a given COS is relevant to the scope of a SR; (2) examine the proportion of SRs for which relevant COS exist; and (3) for SRs for which COS exist, examine the extent to which outcomes in the COS and outcomes in the SR match.

**Study Design and Setting:**

We included a sample of SRs published by the Agency for Healthcare Research and Quality Evidence-based Practice Center Program between January 1, 2018 and October 12, 2020. We searched for potentially relevant COS from the Core Outcome Measures for Effectiveness Trials (COMET) database. We assessed the matching between outcomes recommended by COS and those included in corresponding SRs. When outcomes were matched, we considered matches to be specific (i.e., exact) or general (i.e., non-specific).

**Results:**

Sixty-seven SRs met criteria. We found relevant COS for 36 of 67 SRs (54%). Our framework for comparing the scope of a SR and a COS describes 16 scenarios arising when the breadth of the populations and the interventions are considered. The framework guides systematic reviewers to determine whether a COS is very likely to be relevant, may be relevant, or unlikely to be relevant. Sixty-two percent of outcomes in COS (interquartile range, 40% – 80%) were either specific or general matches to outcomes in SRs.

**Conclusion:**

We found a COS with relevant scope for more than half of the SRs in our sample, with almost two-thirds of the recommended core outcomes matched to outcomes chosen for the SRs. Consideration of COS appears relevant for SR planning and our framework for assessing relevance of a given COS may help with this process.

**What is new?**

**Key findings**
• We found relevant COS for 36 (54%) of a sample of 67 recent SRs.
• A median of 40% of outcomes in SRs (IQR 23% to 56%) were matched to outcomes in corresponding relevant COS.
• We developed a framework to assess the relevance of a core outcome set (COS) to the scope of a systematic review (SR).

**What this adds to what is known?**
• A sizeable proportion of SRs have relevant COS.
• The framework we developed for comparing scope of a given SR and a given COS is new.

**What is the implication, what should change now?**
• Consideration of COS appears relevant for SR planning and our framework for assessing relevance may help with this process.

## 1 Introduction

Systematic reviews (SRs) are conducted to identify and summarize all the evidence addressing a specific research question(s) [1]. For SRs of health-related interventions, systematic reviewers formulate research questions typically by delineating the population, interventions, comparators, and outcomes (PICO) of interest. Outcomes therefore are fundamental to SRs and choice of outcomes is a key issue. Which outcomes are chosen for the SR can greatly impact the conclusions that are made and, consequently, the healthcare, public health, and/or policy decisions that are based on those conclusions [2].

Various considerations are usually made when choosing the specific outcomes for a SR. Some considerations include the relevance of outcomes to decision-making, clinical relevance of outcomes, anticipated likelihood of outcomes being frequently reported in included studies, and desired comparability with related previous SRs. One potential source of relevant outcomes for SRs is existing ‘core outcome sets’ (COS). COS represent minimum sets of outcomes that should be measured and reported in all studies in a given topic area [3].

For systematic reviewers, a particularly attractive aspect of considering existing COS is that COS are usually developed through approaches and principles that are not unlike those used during SR research question development. These include multi stakeholder input, iterative discussions, and consensus generation. Thus, it stands to reason that, when choosing outcomes for the review, systematic reviewers could capitalize on the work done by COS developers and consider outcomes that are recommended in the COS. However, determining whether the COS itself is relevant to a given SR topic is not straightforward. We are not aware of an existing framework to guide researchers in determining whether the scope of a given COS is relevant to the topic of the SR being planned. Even in the scenario where a COS is deemed to be relevant, it remains unclear to what extent the outcomes recommended in the COS and the outcomes chosen for the SR overlap. We therefore conducted an empirical analysis to examine these issues using a sample of recent SRs.

The United States (U.S.) Agency for Healthcare Research and Quality (AHRQ) funds numerous SRs each year through its Effective Healthcare Program [4]. Each SR is conducted by an Evidence-based Practice Center (EPC). There are currently nine EPCs in the U.S [4]. Most SRs conducted under this Program begin with an extensive “Topic Refinement” phase, during which the EPC team engages panels of key informants and technical experts [5]. These stakeholders represent a range of relevant perspectives, such as clinical experts, patients or patient representatives, guideline developers, healthcare payers, regulators, research funders, and others. The Topic Refinement phase involves considerable effort and time (usually 6 to 8 weeks, with multiple iterative group discussions) to refine the planned scope of the SR. Parts of the discussions are focused on identifying and prioritizing relevant outcomes for the planned SR, a process that sometimes involves beginning from scratch.

## 2 Objective

Using recent SRs produced by the AHRQ EPC Program as a case study, our objectives were to: (1) develop a framework to guide the assessment of relevance of the scope of a given COS to a given SR; (2) examine the proportion of SRs for which a relevant COS exists; and (3) for SRs for which a COS exists, examine the type and extent of matching between outcomes recommended in the COS and the outcomes selected for the SR.

## 3 Methods

### 3.1 Selection of systematic reviews

We examined all SRs (either protocols or completed reviews) that were funded by AHRQ, conducted by EPCs, and published on the AHRQ website (https://www.ahrq.gov/research/findings/evidence-based-reports/search.html) between January 1, 2018 and October 12, 2020. We chose to restrict to SRs published in this time period to enable us to examine contemporary practices regarding outcome choice in SRs. Reviews that did not involve assessment of outcomes were excluded.

### 3.2 Identification of relevant core outcome sets

To identify COS that were potentially relevant to the topic area of each SR, we searched the Core Outcome Measures for Effectiveness Trials (COMET) database on October 12, 2020. The COMET database, maintained by the COMET Initiative, is a free, online, regularly updated, searchable database of COS. SRs could be matched to multiple COS and vice versa. One investigator (SLG) assessed the potential relevance of each identified COS to the topic of each SR following an approach used previously (data available on request) [6,7]. Another investigator (IJS) then verified the assessments. Disagreements were resolved through discussion.

### 3.3 Assessing overlap in scope of topics between SRS and potentially relevant COS

We developed a new framework to assess the overlap in scope of topics between SRs and potentially relevant COS. We did this based on discussions among the author team and refined the framework based on feedback received during presentations of the framework to two online webinar audiences: an AHRQ EPC Program quarterly webinar in the U.S. (September 2020) and a COMET Initiative annual webinar, which was international (November 2020). Once we refined the framework, two investigators (SLG and IJS) independently applied it to each pair of SR and potentially relevant COS. Discrepancies were resolved through discussion.

### 3.4 Extracting information and outcomes in systematic reviews and relevant core outcome sets

One investigator (IJS) extracted information about all SRs and the outcomes specified in their Methods sections. For each relevant COS, we used an existing database of previously extracted information regarding participating stakeholder groups and recommended core outcomes. This database includes details of all published COS and was compiled using the data extracted for an annually updated SR of published COS [8], [9], [10], [11], [12], [13].

### 3.5 Matching of outcomes between systematic reviews and relevant core outcome sets

We focused on the outcome domains (the “what,” e.g., quality of life). We did not examine whether the “how” of an outcome (e.g., one particular instrument for measuring quality of life versus another) matched. Because AHRQ EPC Program SRs usually have multiple research questions per SR, we considered matching of outcomes separately for each research question.

We considered a ‘match’ between a particular outcome in a particular research question of a particular SR and a particular outcome in a particular COS to exist if they were generally or specifically related. We defined *general* matches as scenarios in which one outcome was related to the other in non-specific, that is, general terms. For example, for the topic of rheumatoid arthritis, we considered “disease activity” and “joint damage” to be outcomes that were generally matched because disease activity includes joint damage but can also include outcomes other than joint damage. We defined *specific* matches as scenarios in which one outcome was related to the other exactly, for example, “overall survival” and “all-cause mortality.” Our approach to determining the type of match for pairs of outcomes is consistent with an approach that has been described previously [14].

For each pair of outcomes that was generally (i.e., not specifically) matched, we also assessed which of the two outcomes was broader (or narrower). When this could not be determined, we did not make an assessment about the comparative breadth of outcomes.

### 3.6 Statistical analyses

We calculated descriptive statistics (percentages and medians with interquartile ranges [IQRs]) for SRs and relevant COS. We calculated the median percentages of outcomes in each SR research question that were specific matches, general matches, and non-matches with outcomes in each relevant COS. We constructed scatter plots depicting potential relationships between percentage matching (i.e., number of outcomes that were matched out of the total number of outcomes) and the number of outcomes in the research question of the SR and between percentage matching and the number of outcomes in the COS.

We conducted all analyses using Stata Version 16 (College Station, Texas). We estimated the median (and IQR) of the proportion of outcomes that overlap between the COS and SR.

## 4 Results

### 4.1 Included systematic reviews

**Fig. 1:**
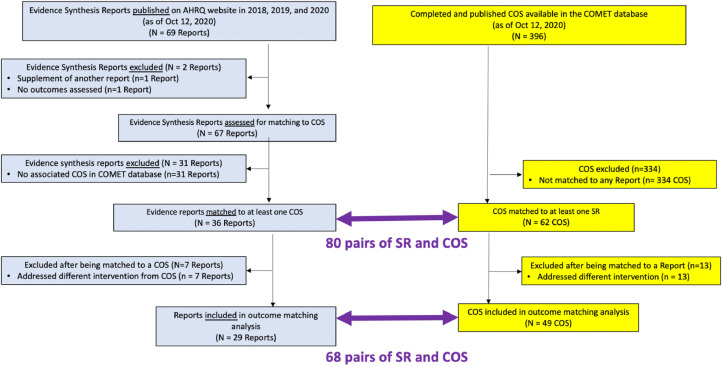
Disposition of systematic reviews (SR) and core outcome sets (COS) in this analysis. (For interpretation of the references to color in this figure legend, the reader is referred to the Web version of this article).

Sixty-nine potentially relevant evidence synthesis reports were published on the AHRQ website between January 1, 2018 and October 12, 2020 (Fig. 1). One report was excluded for not assessing any outcomes and a second report was excluded for being a supplement of another (included) report. We included the remaining 67 SRs.

We found at least one COS with relevant scope for 36 of the 67 included SRs (54%). These 36 SRs were matched to 62 unique COS. However, we excluded seven of the 36 SRs because they addressed interventions that were different from the corresponding COS (see *Framework* section below).

The remaining 29 SRs were included in the outcomes matching analysis and are summarized in Table 1. More than half of the SRs (55%) were published in 2020 and most (86%) were completed versions (i.e., were not protocols). Most SRs focused on treatments (93%) and/or prevention (10%), and most (86%) were comparative effectiveness reviews. SRs excluded from the outcome matching analysis were similar to those we included in terms of years of publications and completion status, but a somewhat smaller proportion of excluded SRs focused on treatments (72%) and/or prevention (4%), or were comparative effectiveness reviews (80%) (data not shown in Table 1).

**Table 1:** Characteristics of the 29 systematic reviews included in the outcomes matching analysis in this analysis

Characteristic	Number of Systematic Reviews (N=29)	
	n	(%)
*Year of publication*		
2018	8	(28)
2019	5	(17)
2020	16	(55)
*Completion status*		
Completed review	25	(86)
Protocol	4	(14)
Focus of review*		
Treatment	27	(93)
Prevention	3	(10)
Diagnostic accuracy	1	(3)
*Type of review*		
Comparative effectiveness review	25	(86)
Topic brief	3	(10)
Technology assessment	1	(4)
*Number of research questions per review (limited to research questions with outcomes)*		
Median	4	
Interquartile range	(3, 5)	
Range	(1, 48)	
Mean	6.0	
Standard deviation	8.9	
*Number of outcomes per research question*+*(limited to research questions with outcomes)*		
Median	8	
Interquartile range	(5, 11)	
Range	(1, 36)	
Mean	10.5	
Standard deviation	8.3	
*Number of core outcome sets with overlapping scope*		
Median	1	
Interquartile range	(1, 5)	
Range	(1, 8)	
Mean	2.3	
Standard deviation	2.0	

⁎ A review could include more than one focus.

+ Across all research questions, regardless of grouping by systematic review.

Included SRs included a median of four research questions (IQR 3 to 5). Research questions included a median of 8 outcomes (IQR 5 – 11).

### 4.2 Framework for assessing overlap in scope between systematic reviews and core outcome sets

**Fig. 2:**
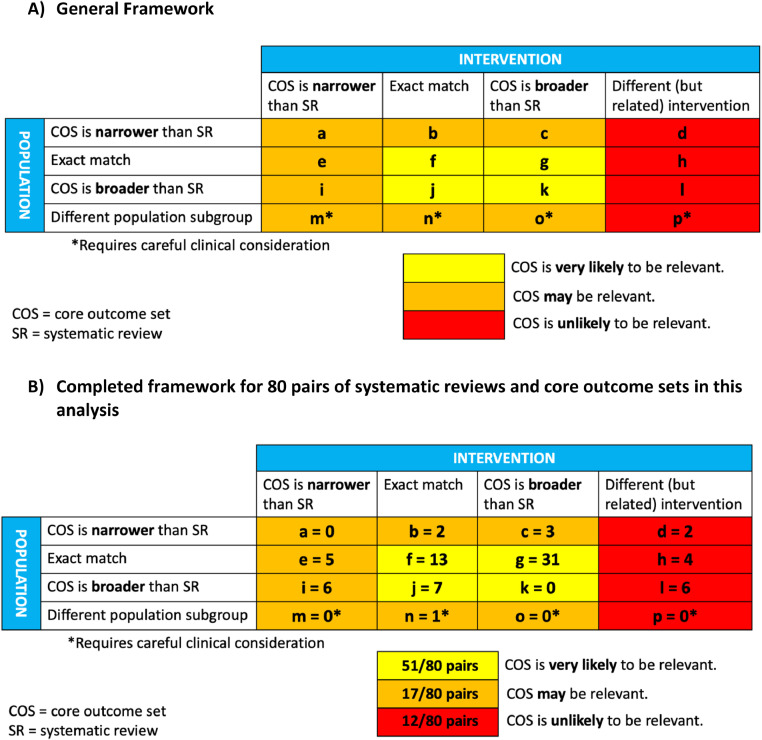
Framework for assessing overlap in scope between a systematic review and a Core Outcome Set (COS). (For interpretation of the references to color in this figure legend, the reader is referred to the Web version of this article).

Fig. 2A provides the new framework we developed for assessing the overlap in scope between a given SR and a given potentially relevant COS. The framework describes various scenarios of degree of overlap in the populations and the interventions examined in pairs of SRs and COS. In terms of the *population*, four scenarios are possible: the COS may be narrower, exact in scope, or broader than the SR, or the COS and SR may describe different subgroups of the population (e.g., adults versus children, adults with mild versus severe cognitive impairment). In terms of the *intervention*, four scenarios are possible: the COS may be narrower, exact in scope, or broader than the SR, or the COS and SR may describe different (but related) interventions. Thus, when considering both population and intervention, 16 scenarios are possible (labeled as ‘a’ through ‘p’ in Fig. 2A). We consider that the COS is *very likely* to be relevant for scenarios in which the COS is at least as broad (i.e., exact in scope or broader) than the SR in terms of both the population and the intervention (scenarios ‘f’, ‘g’, ‘j’, and ‘k’). We consider that the COS *may* be relevant for scenarios in which the COS is narrower than the SR in terms of the population or intervention or both (scenarios ‘a’, ‘b’, ‘c’, ‘e’, and ‘i’) or in which the SR and COS describe different subgroups of the population (scenarios ‘m’, ‘n’, and ‘o’). While all assessments of overlap in scope between a given SR and a given COS should involve clinical expertise for decision-making regarding scope, this is particularly true for scenarios that involve different subgroups of the population (scenarios ‘m’, ‘n’, and ‘o’). Finally, we consider that the COS is *unlikely* to be relevant for scenarios in which the SR and the COS describe different (but related) interventions (scenarios ‘d’, ‘h’, ‘l’, and ‘p’).

### 4.3 Frequency of various scope overlap scenarios in the current analysis

Fig. 2B provides empirical data regarding the frequency of occurrence of all 16 scope overlap scenarios in the current analysis. The figure also includes the 12 instances (from seven SRs) in which the SR and COS described related but different interventions. Thus, Fig. 2B describes the scenarios in all 36 SRs for which we found a potentially relevant COS (i.e., including the seven SRs that are excluded from subsequent analyses). Fig. 2B depicts 80 pairs of SRs and potentially relevant COS. The COS is very likely to be relevant for almost two-thirds of the pairs (51/80; 64%); the COS may be relevant for 17 pairs (21%) and is unlikely to be relevant for 12 pairs (15%). The most frequent scenario (31/80 pairs; 39%) was scenario ‘g’ (i.e., the population was exact in scope, but the COS described a broader intervention or set of interventions). For 13 pairs (16%), both the population and the intervention in the COS and SR were exact in scope (i.e., scenario ‘f’).

### 4.4 Relevant core outcome sets

The 29 SRs for which we found at least one relevant COS (where the interventions were not different) were paired with 49 unique COS. Each SR was assessed to have a median of one relevant COS (IQR 1 to 5) (Table 1). Table 2 describes the 49 COS. Almost a third of the COS (31%) were published between 2016 and 2020, but a quarter (25%) were published before 2006. Various stakeholders participated in the development of the 49 COS. Almost 80% of COS included healthcare professionals, while over half included researchers (57%) and patients (51%). Policy-makers participated in only 8% of COS and guideline developers participated in none. COS included a median of seven outcomes each (IQR 5 to 10).

**Table 2:** Characteristics of the 49 core outcome sets included in the outcomes matching analysis in this analysis

Characteristic	Number of Core Outcome Sets (N=49)	
	n	(%)
*Year of publication*		
2005 or earlier	12	(25)
2006-2010	11	(22)
2011-2015	11	(22)
2016-2020	15	(31)
Stakeholders involved in core outcome set development*		
Healthcare professionals	38	(78)
Researchers	28	(57)
Patients	25	(51)
Policy makers	4	(8)
Guideline developers	0	(0)
Other	15	(31)
*Number of outcomes in core outcome set*		
Median	7	
Interquartile range	(5, 10)	
Range	(1, 19)	
Mean	8.0	
Standard deviation	4.1	

⁎ A core outcome set could include more than one type of stakeholder.

### 4.5 Outcomes in systematic reviews: matches with outcomes in core outcome sets

**Fig. 3:**
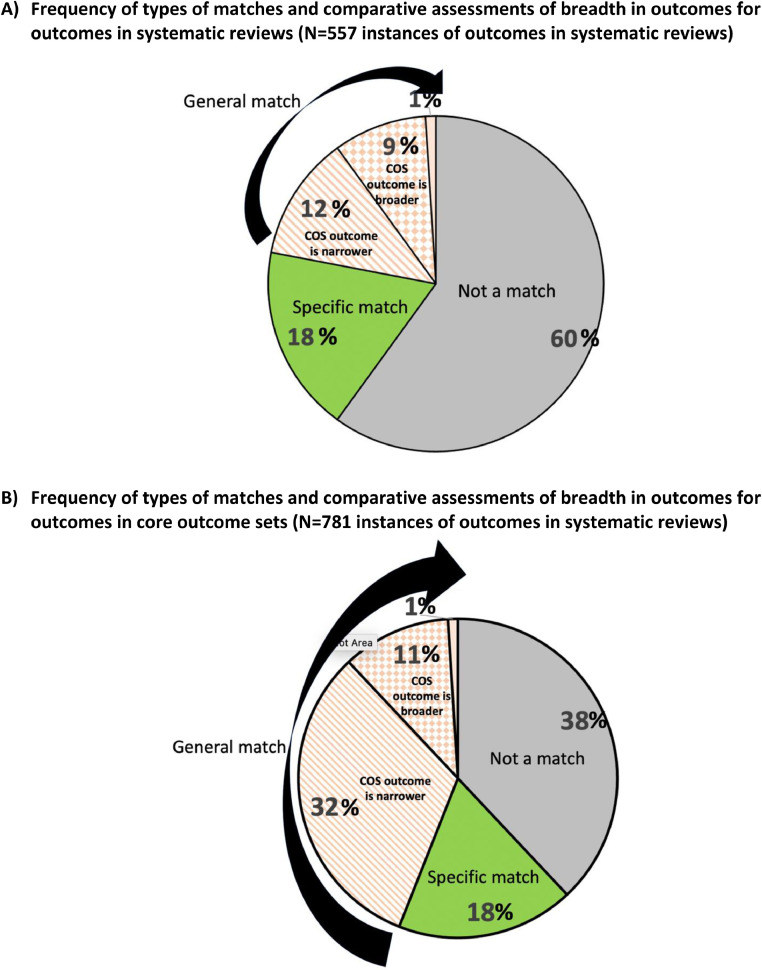
Pie charts. (For interpretation of the references to color in this figure legend, the reader is referred to the Web version of this article).

For the research questions in the 29 SRs, a median of 60% of outcomes per research question (IQR 44% to 77%) were *not* matched with any outcome in corresponding relevant COS (Fig. 3A). A median of 40% of outcomes (IQR 23% to 56%) were either specific or general matches. These included a median of 18% specific matches (IQR 6% to 25%) and 22% general matches (IQR 8% to 33%). Where there was a general match in outcomes (i.e., total of 22%), the COS outcome was narrower in 12%, broader in 9%, and neither in 1%.

**Fig. 4:**
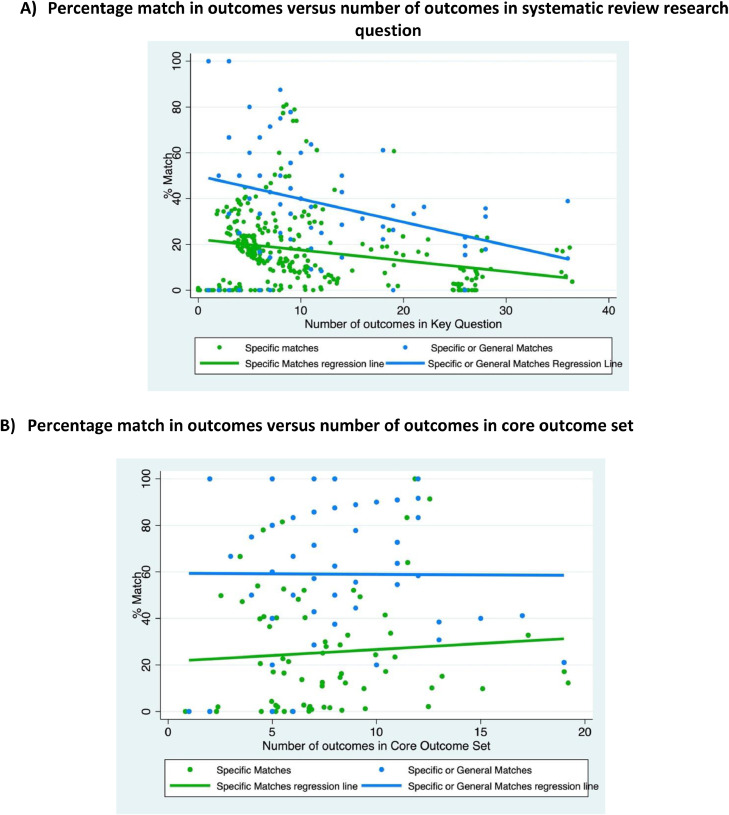
Scatter plots. For Figures 4a and 4b: Each research question and matched COS has two dots, one dot for the percentage of outcomes that are either specific or general matches (blue dot) and another dot for the percentage of outcomes that are specific matches (green). (For interpretation of the references to color in this figure legend, the reader is referred to the Web version of this article).

The scatter plot in Fig. 4A suggests an inverse relationship between the number of outcomes in the research question of the SR and the percentage match between the outcomes in the research question and those recommended in the corresponding COS. This was true for all matches (blue line) as well as for specific matches (green line) in particular.

### 4.6 Outcomes in core outcome sets: matches with outcomes in systematic reviews

For the 49 COS, a median of 38% of outcomes per COS (IQR 20% – 60%) were *not* matched with any outcome in corresponding relevant SRs (Fig. 3B). A median of 62% of outcomes (IQR 40% – 80%) were either specific or general matches to outcomes in corresponding relevant SRs. These included a median of 18% specific matches (IQR 0% – 40%) and 44% general matches (IQR 8% – 60%). Where there was a general match in outcomes (i.e., total of 44%), the COS outcome was narrower in 32%, broader in 11%, and neither in 1%.

The scatter plot in Fig. 4B suggests the lack of a relationship between the number of outcomes in the COS and the percentage match between outcomes in the COS and the corresponding relevant SR. This was true for all matches (blue line) as well as for specific matches (green line) in particular.

### 4.7 Examples of matching and issues encountered

Table 3 provides examples of specific matches, general matches, and non-matches between pairs of outcomes in research questions of the SRs and outcomes recommended in corresponding COS. For generally matched outcomes, Table 3 also includes our assessment of whether the COS outcome was narrower, broader, or neither.

**Table 3:** Examples of specific matches, general matches, and non-matches between outcomes in systematic reviews and corresponding relevant core outcome sets

Systematic review ID	Outcome in systematic review	Core outcome set ID	Outcome In core outcome set	Type of match	Comparative assessment of breadth of outcomes
59	HbA1c	260	HbA1c	Specific	-
59	Hypoglycemia	260	Hypoglycemic episodes	Specific	-
71	Weight loss	247	Weight	Specific	-
50	Haemorrhagic stroke	65	Stroke	General	Outcome in COS is broader.
36	Prostate cancer-specific survival	43	Survival	General	Outcome in COS is broader.
61	Asthma exacerbations requiring systemic (oral and/or parenteral) corticosteroids	60	Exacerbations	General	Outcome in COS is broader.
57	Patient-reported outcomes	29	Pain	General	Outcome in COS is narrower.
59	Quality of life	305	Diabetes-related quality of life	General	Outcome in COS is narrower.
59	Hypoglycemic episodes	305	Number of severe hypoglycaemic events	General	Outcome in COS is narrower.
38	Societal costs, including caregiving time/time spent on activities	95	Family carer burden	General	Outcome in COS is neither broader nor narrower.
89	Asthma-related medication adherence	59	Frequency of medication	General	Outcome in COS is neither broader nor narrower.
61	Asthma exacerbations requiring systemic (oral and/or parenteral) corticosteroids	61	Exacerbations (within 1-4 weeks)	General	Outcome in COS is neither broader nor narrower.
66	Depression	103	Uric acid	Not a match	-
61	All-cause mortality	59	Symptoms	Not a match	-
38	Satisfaction with care	303	Cognition	Not a match	-

Comparisons of breadth of pairs of outcomes were not always straightforward and sometimes led us to assess that, although the outcomes were generally matched, neither the SR nor the COS outcome was broader (or narrower). An example of this is the SR outcome “societal costs” and the COS recommended outcome “family carer burden.” While “family carer” is narrower than the concept of “society,” the concept of “burden” can be broader than “costs” (i.e., burden can also include other aspects, such as emotional burden and physical burden). Table 3 also includes other examples of such scenarios.

## 5 Discussion

### 5.1 Summary of findings

In this case study, more than half (54%) of the recent SRs published by the AHRQ EPC Program were on topics with relevant COS. Forty percent of outcomes in SR research questions were either specific or general matches to outcomes in corresponding relevant COS. Sixty-two percent of outcomes were either specific or general matches to outcomes in corresponding relevant SRs. We developed a new framework to guide systematic reviewers in assessing whether a given COS is relevant to their SR research question.

### 5.2 Scope and other considerations

Although we developed a framework to assess the relevance of a given COS to a given SR in terms of scope, we remind readers that all such assessments should also be informed by clinical considerations that are particular to the topic area. Nevertheless, the elements that constitute the framework, that is., the population and intervention, are fundamental aspects of research questions (PICO) for most health-related SRs. The assessment of whether or not the scope of a COS is narrower than a given SR topic in terms of population (e.g., older adults versus all adults) and intervention (e.g., medications versus any treatment) will usually be straightforward. However, we recognize that the resulting specific determinations of whether the COS is *very likely* to be relevant (scenarios f, g, j, and k in Fig. 2), *may* be relevant (scenarios a, b, c, e, i, m, n, and o), or *unlikely* to be relevant (scenarios d, h, l, and p) may not apply uniformly to all contexts. Our intention with this framework is to provide a guide, but not a rule, for decision-making. This is reflected in the qualifying language used in the framework (i.e., likely, may, and unlikely). We welcome feedback on this framework and an evaluation of its usefulness in various contexts.

Beyond scope, other considerations should also be made when assessing the relevance of a COS to a SR topic. Examples of these considerations include adequacy of the methods of COS development, range and extent of participation of stakeholders, and recency of the COS. Another consideration relates to contexts in which multiple relevant COS exist for a given SR topic. Here, systematic reviewers should pick the COS that is most useful for the SR or select recommended outcomes from more than one of the COS as appropriate.

### 5.3 Numbers of outcomes

The current analysis found an inverse relationship between the number of outcomes in SR research questions and the percentage of those outcomes that were either specific or general matches to outcomes recommended in corresponding COS. This can be explained at least in part by the possibility that systematic reviewers may also be interested in *types* of outcomes beyond those recommended in COS, such as outcomes that directly impact decision-making. In general, there were somewhat more outcomes in the SRs than in the COS (median of eight versus seven outcomes, respectively).

As for outcomes in the COS, the current analysis found no relationship between number of outcomes and the extent of their overlap with SR outcomes. This finding is consistent with a recent SR by Hughes and colleagues that found no evidence of a relationship between number of outcomes in COS and uptake of the COS [15]. In that SR, the COS with the highest level of uptake recommended seven outcomes.

### 5.4 Implications for systematic reviewers

Outcome heterogeneity across included studies continues to be a problem across topics for SRs, being explicitly noted in 40% of recent Cochrane SRs [7]. In another analysis, authors of 41% of recent Cochrane Eyes and Vision SRs could not conduct a meta-analysis for the SR's primary outcome because fewer than two included studies measured that outcome or the specific measurements (i.e., measurement instruments or definitions) for the outcome were inconsistent across studies [2]. To help alleviate some of these challenges, we advocate more widespread uptake of COS by systematic reviewers. While COS have traditionally been developed for use in clinical trials, considerations regarding scope and contextual relevance notwithstanding, there is no obvious reason why outcomes recommended in COS should not be considered for SRs. Although, in the current analysis, the percentage of SR outcomes that are also recommended in corresponding COS was only 40%, outcomes in COS could provide a starting list of outcomes for consideration during the early stages of a SR. Then, the SR team could consider additional outcomes that are not in the COS. Another way in which COS can be useful for systematic reviewers is for the COS-recommended outcomes to serve as prioritized outcomes for the Summary of Findings tables [2,16]. Such tables are intended to convey the key findings of the SR.

Our search of the COMET database on April 7, 2021 revealed 406 published COS for research and a further 360 under development. Taken together, these COS cover a range of clinical topic areas, although the numbers of COS within topic areas vary. The COMET database is online, free, and easily searchable. Among the various purposes of the COMET database is the reduction of research waste that can occur due to potential unnecessary duplication of effort [16,17]. We encourage systematic reviewers to capitalize on the COMET database resource and search it when choosing outcomes for the SR. This is consistent with current guidance from Cochrane for those conducting Cochrane SRs [1]. We also encourage the curators of SR protocol registries, such as PROSPERO, to inform (or remind) authors of SRs to search the COMET database for relevant COS. Ideally, this should happen while the SR protocol is being registered.

### 5.5 Implications for the ‘Evidence Ecosystem’

Schünemann and colleagues recently conceptualized a Standardized Outcomes Linking Across Stakeholders (SOLAR) system, in which various kinds of decision-makers may use outcomes for different purposes, but they should all link to patient-centered outcomes [18]. In such a system, randomized controlled trial and other research outcomes may not be substantially different from SR outcomes. The current analysis suggests that approximately 40% of SR outcomes are also recommended by relevant COS. While this percentage may suggest some good news in terms of concordance between outcomes recommended in COS and outcomes reported in SRs, the percentage is not very high. Prior work has suggested that clinical trialists and systematic reviewers might not always be interested in the same types of outcomes. For example, compared with clinical trialists, systematic reviewers may be interested in more patient-centered and long-term clinical outcomes [19]. We agree with Schünemann and colleagues that the “*health care space is broader than clinical research*” and that relevant stakeholders should “*harmonize outcome assessment for the different decision-making contexts*.” [18]

In the current analysis, we did not explore (1) whether the systematic reviewers were aware of the availability of relevant COS and (2) for those that may have been aware, their reasons for not including outcomes from the COS in the review (or why they may have changed their plans to do so). Although these questions were beyond the scope of the current analysis, future research should explore them.

Nevertheless, we believe that more widespread adoption of COS in the evidence ecosystem is likely to happen in the future. To help ensure that the most relevant outcomes are incorporated into COS (and eventually into studies that are included in SRs), we agree with the suggestion that more systematic reviewers should become involved in COS development [2,16]. One example of a formal initiative to promote such involvement is the Cochrane Skin Core Outcome Set Initiative [20]. Relatedly, the Red Hat Group is a network established with the intention of bringing together various organizations interested in the development and adoption of COS in the evidence ecosystem [21].

### 5.6 Limitations

The current analysis has some limitations. First, the SRs analyzed were from one organization (AHRQ) and were mostly comparative effectiveness reviews of treatments or preventive interventions. They may thus not be representative of all SRs. It is possible that our findings would have been different had we examined a different sample of SRs. However, perhaps unsurprisingly (because COS have traditionally been developed for clinical trials), a large proportion of published COS are about treatment and/or preventive interventions. Second, the COS in the current analysis were developed with limited participation of policymakers (8%) and guideline developers (0%). It is possible, perhaps likely, that the degree of matching between outcomes in COS and SRs examined in the current analysis would have been higher had these stakeholders participated in the development of the COS examined.

## 6 Conclusions

In summary, we found a COS with relevant scope for more than half of the SRs in our sample, and more than 60% of core outcomes are reported in corresponding relevant SRs. We developed a framework for assessing the scope of a given COS in relation to the topic of a given SR. We encourage systematic reviewers to consider COS when choosing outcomes for the review and to get more involved in COS development efforts.

## Contributorship statement

*Study concept and design*: Saldanha and Williamson. *Acquisition of data*: Saldanha, Dodd, and Gorst. *Analysis and interpretation of data*: All authors. *Drafting of the manuscript*: Saldanha. *Revising manuscript for intellectual content*: All authors. *Final approval of the completed manuscript*: All authors.

## Ethics Committee Approval

Not applicable.

## Clinical Trials Registration

Not applicable.

## Data Sharing Declaration

Data will be shared upon request.
